# Chronic Traumatic Encephalopathy in Contact Sports: A Systematic Review of All Reported Pathological Cases

**DOI:** 10.1371/journal.pone.0117338

**Published:** 2015-02-11

**Authors:** Joseph C. Maroon, Robert Winkelman, Jeffrey Bost, Austin Amos, Christina Mathyssek, Vincent Miele

**Affiliations:** Department of Neurosurgery, University of Pittsburgh Medical Center, Pittsburgh, Pennsylvania, United States of America; UCL Institute of Neurology, UNITED KINGDOM

## Abstract

Chronic traumatic encephalopathy (CTE) is a neurodegenerative disease associated with head trauma. Although initially believed to affect only boxers, the at-risk population has expanded to encompass a much wider demographic, including American football players, hockey players, wrestlers, and military veterans. This expansion has garnered considerable media attention and public concern for the potential neurodegenerative effects of head trauma. The main aim of this systematic review is to give a complete overview of the common findings and risk factors for CTE as well as the status quo regarding the incidence and prevalence of CTE. This systematic review was performed using PubMed and MEDLINE and includes all neuropathologically confirmed cases of CTE in the medical literature to date, from the first published case in 1954 to August 1, 2013 (n = 153). The demographics, including the primary source of mTBI (mild Traumatic Brain Injury), age and cause of death, ApoE genotype, and history of substance abuse, when listed, were obtained from each case report. The demographics of American football players found to have CTE are also presented separately in order to highlight the most prevalent group of CTE cases reported in recent years. These 153 case reports of CTE represent the largest collection to date. We found that a history of mTBI was the only risk factor consistently associated with CTE. In addition, we found no relationships between CTE and age of death or abnormal ApoE allele. Suicide and the presence of premorbid dementia was not strongly associated with CTE. We conclude that the incidence of CTE remains unknown due to the lack of large, longitudinal studies. Furthermore, the neuropathological and clinical findings related to CTE overlap with many common neurodegenerative diseases. Our review reveals significant limitations of the current CTE case reporting and questions the widespread existence of CTE in contact sports.

## Introduction

The damaging neurological effects of sports-related repetitive head trauma were described by Harrison S. Martland in 1928 [1]. His clinical description of ‘punch drunk syndrome’ in a group of former boxers has been extended to include a complex neuropathological and clinical diagnosis known today as Chronic Traumatic Encephalopathy (CTE). More recently case reports demonstrate pathologically confirmed CTE in former combat military personnel and contact sport athletes other than boxers. This has prompted renewed interest and controversy regarding the potential for long-term neurodegenerative changes to occur after concussive and even sub-concussive repetitive or blast wave associated head trauma [2, 3].

Thus far CTE research has been limited to selective case reports. There are no published systematic studies incorporating both sport and non-sport related head trauma populations. Based on this lack of data, it is currently impossible to determine the incidence of new cases occurring within contact sport. Additionally, overall prevalence of CTE amongst all cases of head trauma cannot be determined at this time. Finally, due to the fragmented data collected in case reports, no conclusions can be drawn about potential risk factors for developing CTE in contact sports [4]. To date, all pathologically confirmed CTE cases have had a history of head trauma; however, the reported degree of severity, frequency of blows to the head, and documentation of prior concussion is highly variable [5].

Several recent reviews have focused on the various neuropathological findings and the clinical criteria used for the diagnosis of CTE and have drawn attention to the confusion and inconsistency of the diagnosis of CTE [6–7]. These reviews have highlighted confounding neuropathological findings such as the presence of co-occurring neurodegenerative conditions including Alzheimer’s disease, Parkinson’s disease and Amyotrophic lateral sclerosis (ALS) discovered at the time of death in those reported to have CTE.

In this review, we summarize all currently known cases of pathologically-confirmed CTE, from the first published case in 1954 to August 1, 2013. Our purpose is to provide a data-driven overview of all CTE cases to date. The demographics, including the primary source of mTBI (mild Traumatic Brain Injury), age and cause of death, ApoE genotype, and history of substance abuse, when listed, were obtained from each case report. The demographics of American football players found to have CTE are also presented separately in order to highlight the most prevalent group of the many CTE cases reported in recent years. Through this review we will describe the limitations of current CTE case reporting in order to place into context the lack of evidence for the widespread existence of CTE in contact sports.

## Methods

We reviewed all known cases of pathologically confirmed CTE from the first published case in 1954 to August 1, 2013 using the online databases of PubMed, MEDLINE, and Google Scholar. Non-English language articles were included and translated. Initially, articles were identified using a combination of the following search terms: "chronic traumatic encephalopathy", "dementia pugilistica", "traumatic brain injury", "repetitive mTBI", “sports-related concussion”, "neurodegenerative disease", "hyperphosphorylated tau", "tauopathy", “TDP-43 proteinopathy”, “professional football”, “boxing”, “military veterans". Based on the abstracts, all studies that described neuropathological reports of CTE and DP were selected for review. In addition, all references from the selected articles were searched and any relevant references were also included in the review. Information from media reports was also screened for confirmed CTE cases. The subjects compiled in this review consist of pathologically confirmed cases of DP/CTE reported in the medical literature, and four subjects whose information was gathered from confirmed media reports. Exclusion criteria included: only presenting clinical findings of suspected head trauma-related neurodegeneration without post-mortem neuropathological confirmation, subjects not explicitly diagnosed with either DP or CTE, and duplicate cases republished in the medical literature, which were identified either by explicit flagging of the authors or by matching demographic information from previous studies by the same author.

### Measures

Individuals were categorized according to the most likely source of potential head impact. Categories were: Boxer (amateur or professional), American football player (national football league (NFL), Canadian football league (CFL), semi-professional (semi-pro), collegiate football (collegiate), and high school (HS), ice hockey player (amateur or professional), wrestler (amateur or professional), military veteran or miscellaneous. If an athlete was also a military veteran, they were still categorized according to their sport involvement. Additional potential causes of head trauma, such as motor vehicle accident (MVA) and fights were also included when reported.

Age at death was included and classified according to a range over 10 years (i.e. 10–19, 20–29, etc.). Specific cause of death was also reported when available and was additionally classified as natural, accidental, or suicide.

Apolipoprotein (ApoE) genotype profiling was included if the original source provided the information. History of substance abuse was dichotomized (yes; no/unknown) based on information deducted from case reports and media reports, using substance abuse criteria from the American Psychiatric Association's Diagnostic and Statistical Manual of Mental Disorders (DSM-IV) [8] as guideline. Hence, individuals identified as having a positive history of substance abuse were not necessarily clinically diagnosed.

## Results

**Fig 1:**
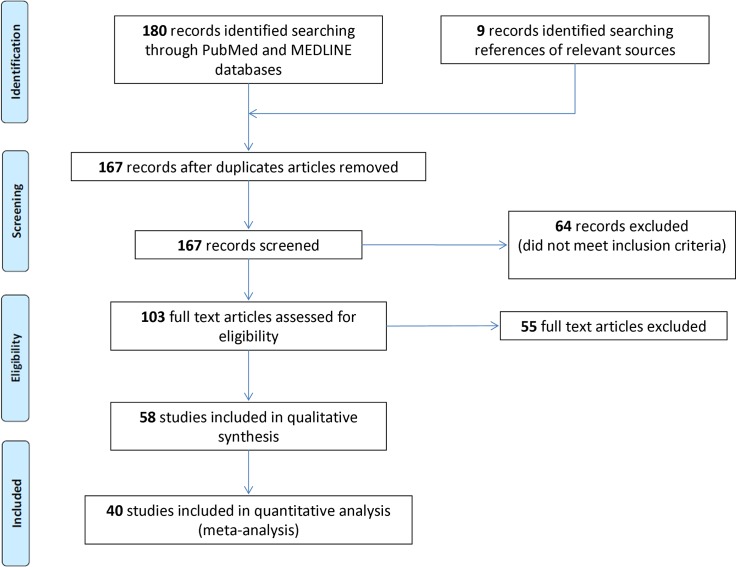
Prisma flowchart illustrating the number of included and excluded studies in the systematic review on Chronic Traumatic Encephalopathy in Contact Sports.

A literature review of 180 sources rendered 40 articles reporting on one or more neuropathologically confirmed cases of CTE [2,3,5,9–45] (Fig. 1). This review revealed a total of 262 confirmed cases of CTE. Of these 262 cases, 113 were determined to be duplicates, resulting in a total of 149 unique cases with pathologically confirmed CTE in the medical literature. In addition to these 149 cases, an additional 4 cases of CTE were identified in published media reports that were not also included in articles of the literature review [46–49]. A final cohort of 153 unique pathologically confirmed cases of CTE/DP was determined from combined literature and media reports data.

The 153 cases of CTE (S1 Table) included n = 69 (45.1%) former boxers, n = 63 (41.2%) former amateur and professional football players, n = 5 (3.3%) former hockey players, n = 6 (3.9%) former military veterans, n = 3 (2.0%) former professional wrestlers, and n = 7 (4.6%) miscellaneous cases [30–61]. Some of the former athletes are also military veterans (n = 15 former football players, n = 11 former boxers, and n = 1 former hockey player). With one exception, all diagnosed cases with CTE have been males.

The age of death was available for 150 cases and ranged from 17 to 98 years. Individuals 60–69 years of age made up the largest age demographic in this series (Table 1). Information about the cause of death was available for 111 cases. There were 78 deaths from natural causes; 19 accidental deaths, and 14 suicides. Common causes of natural death were respiratory failure, cardiac disease, failure to thrive associated with end-stage dementia, and malignancy. Common causes of accidental death were drug overdose and severe TBI injury.

**Table 1:** Age group distribution in CTE diagnosed subjects.

**Age range**	**Overall cases n (%)**		**Football cases n (%)**	
10–19	3	(2.0%)	3	(4.8%)
20–29	16	(10.7%)	5	(7.9%)
30–39	9	(6.0%)	6	(9.5%)
40–49	21	(14.0%)	11	(17.5%)
50–59	21	(14.0%)	6	(9.5%)
60–69	39	(26.0%)	13	(20.6%)
70–79	26	(17.3%)	10	(15.9%)
80–89	12	(8.0%)	8	(12.7%)
90–99	3	(2.0%)	1	(1.6%)
Total	150		63	

ApoE allele genotyping data was available for 80 cases (Table 2). Of these, homozygous ApoE3 (n = 49; 61.3%) and heterozygous ApoE3/E4 (n = 20; 25%) were the most prevalent genotypes. Other genotypes such as Apo E2/E3 (n = 4; 5.1%), E2/E4 (n = 2; 2.5%), and E4/E4 (n = 5; 6.3%) were observed at much smaller frequencies.

**Table 2:** ApoE genotype breakdown in CTE diagnosed subjects.

**ApoE genotype**	**Overall cases n (%)**		**Football cases n (%)**		**% of normal population**
ε3/ε3	49	(62.0%)	32	(60.4%)	58.5%
ε2/ε3	4	(5.1%)	4	(7.5%)	13.6%
ε2/ε2	0	(0.0%)	0	(0.0%)	0.3%
ε2/ε4	2	(2.5%)	1	(1.9%)	2.4%
ε3/ε4	20	(25.3%)	11	(20.8%)	22.2%
ε4/ε4	5	(6.3%)	5	(9.4%)	2.9%
Total	80		53		

Thirty (19.6%) cases had a documented history of substance abuse prior to or co-morbid with symptomatic CTE presentation (Tables 3, 4). Substances abused included alcohol, prescription painkillers, anabolic steroids, cocaine, methamphetamine, and marijuana.

**Table 3:** History of substance (ab)use in CTE diagnosed subjects.

**Substances (ab)used**	**Number of cases**	**% of all substances abuse**
Alcohol	20	71.4%
Painkillers (Opioids/Opiates)	5	17.9%
Cocaine	4	14.3%
Marijuana	2	7.1%
Methamphetamines	2	7.1%
Anabolic Steroids	4	14.3%

**Table 4:** History of substance (ab)use by primary TBI exposure groups.

**Primary TBI exposure**	**Number of cases**	**% of respective group**
Football	9	14.3%
Boxers	13	18.8%
Hockey	3	60.0%
Wrestling	2	66.7%
Veterans	2	33.3%
Other	1	14.3%
Total	30	19.6%

There are inherent limitations on the numbers reported for substance abuse. The information gathered commenting on substance abuse was often a result of post mortem interviews from next of kin members who were likely unaware of the extent of abuse. Also substance abuse problems are difficult to diagnose and may go undetected by family members. Also, not able to gather information regarding the extent and frequency of substance abuse.

Since 2002, the majority of CTE cases documented in the literature have involved American football players. Now totaling 63 subjects, football players are second only to boxers in terms of documented CTE diagnoses in the medical literature. Of the 63 former football players, 6 played only high school level and 9 through the collegiate level. At the professional level, 42 played in the National Football League (NFL), 4 the Canadian Football League (CFL), and 1 played semi-professional football. Age of death was available for all 63 players, with the most common age range of death being 60–69 years (Table 1). Cause of death was also available for all 63 football player, with n = 41 dying from natural causes (65.1%), n = 11 from accidental causes (17.5%), and n = 11 from suicide (17.5%). ApoE allele genotyping data was available for 53 out of 63 of the football players (84.1%; Table 2). A positive history of substance abuse was found in 9 football players (14.3%; Table 4).

## Discussion

This review of 153 pathologically confirmed cases of CTE represents the most current and most complete number of confirmed CTE cases in the medical literature. The final number of CTE cases was determined after accounting for 113 duplicate reported cases. Duplicate cases accounted for 43% of all cases of CTE identified in the medical literature by this review. Although the authors of this review acknowledge the occasional need for re-evaluating former CTE cases in order to further understand CTE findings presented to date, the high rate of re-reporting cases often without explicit notation of previous documentation has led to an erroneous, inflated impression of the number of CTE cases reported. The 153 CTE cases described in this review also include four unique cases of CTE found in media reports which were substantiated by cross confirmation from multiple sources including quotes from CTE investigators [46–49].

Of the 153 unique pathologically confirmed cases of CTE, six major mTBI subgroups were identified: former boxers, former football players, former hockey players, former military veterans, former professional wrestlers, and other miscellaneous causes of head trauma. Former boxers and football players made up the majority of all cases (86.2%). This observation is consistent with the long standing history of CTE research in the sport of boxing and the recent focus on former football players.

Starting in 1954 the pathological diagnosis of CTE, first recognized in a former boxer, remained exclusive to this sport for 35 years until it was observed in a non-boxing subject in 1989 [11,40]. The modern interest in CTE began in 2002 with the seminal diagnosis of CTE in a former professional football player by Dr. Bennett Omalu [35]. Since then, football has been the most studied mTBI subgroup for CTE accounting for 63 cases compared to boxing’s 17 during the same time period. The remaining mTBI subgroups identified since 2002, including former combat military personnel and other contact sport athletes such as ice hockey players, have yet to be studied to the same extent as either boxers or football players. This is also true for athletes of contact sports with risk for head impact but with no confirmed CTE cases to date, such as rugby or soccer players. Based on the current state of CTE research, we have no reason to hypothesize that the clinical and pathological presentation of CTE in other contact sports would systematically differ from the CTE presentation in boxers or football players; yet, more systematic research data is necessary to evaluate this hypothesis.

Despite media speculation and several prominent CTE research groups purporting to estimate the prevalence of CTE in former boxers and football players, there has not yet been a large, longitudinal study conducted to substantiate any estimates [50,51]. The number of former participants in contact sports and military combat with a reported history of mTBI is estimated to be in the millions and to determine the actual incidence and prevalence of CTE would require thousands of participants and would need to span decades. The 4^th^ International Conference on Concussion in Sport in 2012 also concluded the prevalence and causation of CTE cannot be inferred at this time [4] (see Consensus Statement below).

### 2012 Consensus Statement on Concussion in Sport: *Chronic traumatic encephalopathy*

“*Clinicians need to be mindful of the potential for long-term problems in the management of all athletes*. *However*, *it was agreed that chronic traumatic encephalopathy (CTE) represents a distinct tauopathy with an unknown incidence in athletic populations*. *It was further agreed that a cause and effect relationship has not as yet been demonstrated between CTE and concussions or exposure to contact sports*. *At present*, *the interpretation of causation in the modern CTE case studies should proceed cautiously*. *It was also recognized that it is important to address the fears of parents/athletes from media pressure related to the possibility of CTE* [4].”

**Cause and age of death**. The age of death reported in 150 CTE cases ranged from 17 to 98 years old, with the median age of death falling in the range of 60 to 69 years. This represents an earlier average age of death compared to the general US male population age of death of 76.2 years [52]. Of the CTE cases, 72.7% died before the age of 70. Of the 111 CTE cases that included cause of death, none listed CTE as cause of death. The majority of the CTE subjects died from natural causes; however, the prevalence of suicide (11.7%) and accidental deaths (17.5%) is much higher in the CTE population than in the general population (1.5% and 4.8%, respectively) [52]. The greater percent of suicides and accidental deaths in the CTE case reports thus far have likely contributed to the lower average age of death in this selected group.

Suicide and accidental death are a more recent observation reported in subjects with CTE, with all suicides and 70% of accidental deaths coming after 2002. Researchers have suggested that reported CTE related symptoms such as lack of impulse control, criminal and antisocial behaviors, and mood disorders may be associated with the increased prevalence of suicide and accidental deaths [33]. A review by Iverson (2014), however, points out that suicide and accidental death have been reported more often in less advanced stages of CTE thereby suggesting that such behaviors may not be due to progression of CTE [53]. Selection-bias due to the greater CTE reporting on high profile former football players who have committed suicide or died due to accidental death has contributed to a possible overestimate of this cause of death. Published reports have noted both individuals and families of players who have died by suicide or accidental death have been disproportionately more likely to participate in CTE brain donation programs [2].

**Neuropathology**. Despite the younger average age of death in the CTE population compared to the general population, the typical age of death of subjects diagnosed with CTE can be considered advanced; hence, the effects of other age-related neuro degeneration diseases must also be taken into account [54,55]. The general pathological characteristics associated with CTE include macroscopic degenerative changes such as cavum septum pellucidum, generalized global atrophy, thinning of the corpus callosum, and ventricular dilatation. Neurofibrillary tangles similar to AD are seen but not the density of senile plaques which are generally observed in AD. Irregular, multifocal, and generally perivascular tau-immunoreactive neurofibrillary tangles (NFTs) are now considered a pathognomonic finding exclusive to CTE distinguishing it from other neurodegenerative diseases [3]. Beta-amyloid (Aβ) deposits are only identified in about 40% of those found to have CTE, as compared to extensive Aβ deposits present in nearly all those pathologically confirmed to have AD [5].

Large population studies reviewing pathological findings in the general population have shown that neurodegenerative pathological changes similar to CTE can occur naturally with the aging process. In a post mortem study with a non-selected cohort of 2661 subjects, Braak et al. reported that 91.6% of the subjects over the age of 60 had evidence of neurofibrillary tangles (NFT) [56]. Braak et al. as well as others have reported tau pathology findings in much younger cases, albeit at much lower frequencies compared to elderly subjects. In addition to tau related pathology, abnormalities in the transactive response (TAR) DNA binding protein with molecular weight of 43-kDa (TDP-43) have also been observed postmortem in the brains of CTE cases [26]. TDP-43 pathology is a proteinopathy similar to tauopathy that is commonly observed in other neurodegenerative diseases such as in frontotemporal lobar degeneration (FTLD) and ALS. The findings of natural age-related pathology including both tau and TDP-43 have been been commonly observed in elderly non-demented controls in a number of studies as well [57–59].

**Age**. Increasing age is also a major risk factor for other neurodegenerative diseases such as Alzheimer’s disease (AD), Parkinson Disease (PD), Lewy body dementia (DLB), and cerebrovascular disease (CVD) [55]. McKee et al. 2013 reported that the 20 oldest subject of their 68 CTE cohort also displayed CTE-related neurodegenerative changes consistent with AD, PD, and DLB among other diagnoses [2]. Although the interaction between diseases is unknown, one cannot discount that the presence of a co-morbid condition may distort both the clinical and pathological picture associated with CTE. As stated previously, CTE shares numerous neuropathological abnormalities commonly associated with other neurodegenerative diseases. Consequently, it is unclear what is responsible for specific clinical changes currently attributed to pre-morbid CTE [22,32]. Omalu et al. (2011) stated that caution should be exercised when diagnosing CTE in those over the age of 65 since normal age related changes in the brain, Alzheimer’s disease, chronic ischemic changes and small vessel disease in the brain could lead to confusion with pathological CTE related changes.

Smith et al. [6] has proposed the pathogenesis of CTE may be best described as an interaction between neuropathological changes due to head trauma and natural age related changes of the brain. The exact contribution of prior head trauma on the clinical and neuropathological changes currently associated with CTE remains unclear and clinical symptoms may be a consequence of multiple neurodegenerative conditions including CTE. Importantly, the presence of neurodegenerative changes is not always associated with clinical symptomatic presentation [6].

Blaylock and Maroon postulated that immunoexcitotoxicity is a central mechanism in the development of CTE [60]. Based on this concept, microglia, which are activated following TBI, initiate injury response mechanisms resulting in the release of various cytokines, chemokines, reactive oxygen and nitrogen species as part of the innate inflammatory response. This is followed by a reparation and regeneration process—provided there is no recurrent blows to the head. With repetitive brain trauma, before the reparative process, immune mediators along with the traumatic release of excitatory intracellular glutamate can trigger a cascade of neuronal damage including dendritic retraction, synaptic injury, damage to microtubules, mitochondrial suppression and the over-production of tau.

Omalu et al. (2011) observed in an 18-year-old former high school football player “none to sparse” neurofibullary tangles in the cerebral cortex, subcortical nuclei, and brain stem [3]. However he did conclude there was sufficient evidence of CTE but described these pathological findings as “incipient CTE”, and suggested such findings potentially represented an initial stage of CTE development. McKee et al. have described a CTE pathology staging system to standardize CTE findings. Stage I lists the earliest abnormalities observed for CTE as focal epicenters of pathological tau located in the frontal cortex [2]. By contrast, in more advanced CTE Stages III-IV the neuropathology is characterized by less focal and more widespread distribution of pathological tau throughout the cerebrum, subcortical nuclei, brainstem, and spinal cord. These neuropathologies are typically observed in older CTE subjects [2]. McKee et al. has also reported a correlation of greater neuropathologic changes with greater number of years played, thus suggesting more advanced CTE pathology is linked to increased exposure to head trauma, and exclusive to older age of death. Due to limited available data the natural progression of CTE is still unknown. Whether less-advanced cases of CTE reported in generally younger subjects would eventually progress to advanced neuropathologic findings associated with advanced CTE has not been documented. There are early reports that PET scanning for tau protein may be useful to determine premorbid CTE and could possibly be used to follow changes over time [61].

**Apolipoprotein E**. Apolipoprotein E (ApoE) allele is a well-known genetic risk factor for Alzheimer disease. Kutner et al. and Jordan et al. have also reported that the presence of ApoE4 allele is associated with worse cognitive deficits in patients following severe head injury [62,63]. Data on ApoE genotype was available for 80 CTE cases, which represents the largest compilation of CTE ApoE genotype to date. A Chi-square goodness of fit test was used to compare ApoE genotype of this CTE population to the ApoE genotype of the general population (data adapted from Beydoun et al.) [64]. This analysis revealed no significant group difference between ApoE4 carriers in the CTE population and the general population (p = .26), suggesting that ApoE may not be a significant risk factor for the development of CTE. The result of this analysis is similar to findings by McKee et al. (2012) who also reported non-significant difference between ApoE genotype of the general population and the 68 cases of CTE in presented in their cohort [2]. The non-random selection and incomplete data of all CTE subjects limits speculation as to the significance of these findings. Further comparisons are needed to investigate the differences between post-mortem ApoE genotype of CTE, mTBI survivors and the general population.

**Substance abuse**. Thirty cases (20%) out of 153 CTE cases reported a positive history of substance abuse, which is greater than the 7.7% reported by Comptom et al. for substance abuse in US adults over their lifetime [65]. Cottler et al. (2011) reported that 52% of retired football players used opioids during their NFL career, of which 71% reported misuse [66]. CTE researchers have noted that the clinical presentation of CTE may be distorted due to history of substance abuse [2]. Research investigating neurodegenerative effects of substance abuse has shown that several commonly abused substances may potentiate the development of other neurodegenerative conditions [67–75]. These changes can include tau pathology, activated microglia, neuronal loss, and white matter rarefaction. In fact, in addition to these non-specific neurodegenerative changes, abuse of some substances such as opiates and opioids may elicit pathology very similar to those observed in CTE. In their research detailing the postmortem findings of opiate and opioid abusers, Anthony et al. (2010) observed enhanced NFT and NT deposition in their drug user subjects when compared to normal age-matched controls [74]. Notably, histopathological analysis revealed the enhanced tau pathologies observed in the drug users were analogous to the pathological tau found in AD and CTE, but as in the case of CTE, the distribution was determined to be distinct from classical AD progression. How substance abuse may influence, co-present, or alter the pathologies observed in CTE is unknown. Due to the limited number of CTE cases that include information about history of substance use/abuse as well as limited reporting on the extent of use/abuse, no conclusion can be made as to the presence and nature of an association between substance abuse and CTE.

**CTE related clinical signs and symptoms**. Due to a lack of consistent clinical data in the CTE case reports, this review did not specifically discuss the various premorbid clinical signs and symptoms reported with CTE. Reported CTE-related sign and symptoms include headache, difficulties with attention and memory, mood disorders, motor dysfunction and dementia [2,3]. McKee et al. have proposed a classification system (Stage I-IV) correlating advanced clinical findings to the degree of expected post mortem neuropathologic changes found with CTE [2]. (Table 5) For comparison, Table 5 includes the clinical symptoms of CTE as well as clinical symptoms of other common neurodegenerative conditions. This comparison demonstrates a significant overlap between signs and symptoms of CTE and other common neurodegenerative conditions. Based on this proposed classification it is difficult to distinguish symptomatic CTE from other neurodegenerative diseases such as Alzheimer's disease (AD), Parkinson's disease (PD), and FTLD. Postmortem neuropathological assessment remains the standard for definitive diagnosis [2,3,6,76].

**Table 5:** Clinical symptoms of CTE and other neurodegenerative conditions.

	**CTE (stages)**				**Presence in other neurodegenerative conditions**			
Symptoms	I	II	III	IV	PCS	AD	PD	FTLD
Asymptomatic	x	x	x					
Headache	x	x	x	x	x			
Attention/Concentration loss	x	x	x	x	x	x	x	x
Short-term Memory loss	x	x	x	x	x	x	x	x
Mood Disorder	x	x	x	x	x	x	x	x
Behavioral Problem	x	x	x	x		x	x	x
Executive Dysfunction	x	x	x	x		x	x	x
Language Difficulties	x	x	x	x		x	x	x
Visuospatial Difficulties			x	x			x	
Cognitive Impairments	x	x	x			x	x	
Suicidality		x	x	x		x		
Dementia			x	x		x	x	x
Motor Impairments		x	x	x		x	x	x

Table 5 also compares clinical findings currently attributed to CTE to common signs and symptoms seen with post-concussion syndrome (PCS). In most cases, signs and symptoms associated with a concussion resolve within 1–4 weeks. PCS is characterized by a protracted course following the incident of mTBI that may last months to years following injury [77]. Reynolds et al. and others have demonstrated that over time therapeutic interventions, such as cognitive, visual and balance therapies can improve recovery rates in patients with persistent PCS [78]. Based on McKee’s proposed clinical classification, symptoms of PCS substantially overlap with incipient symptoms of CTE. But unlike PCS where symptoms continue well after an mTBI, CTE attributed clinical findings are generally recognized as having a latent asymptomatic period of years to decades between exposure to mTBI and CTE symptom onset. Additionally, CTE, once symptomatic, is generally thought to be progressive and does not resolve over time [5]. Despite the differences in the general onset of these conditions, some researchers have reported PCS proceeding directly to pathologically diagnosed CTE, further skewing the clinical diagnosis of CTE [79].

Most cases of neuropathologically confirmed CTE have reported signs and symptoms of neurological decline prior to death. However, McKee et al. (2012) reported 11% of their CTE-positive cohort (n = 5) were asymptomatic for any neurological conditions. Asymptomatic presentation was reported as advanced as CTE Stage III where there is widespread NFT pathology and neurodegeneration [3]. Although asymptomatic presentation of neuropathology such as in AD is common, it remains unclear why some pathologically diagnosed CTE subjects experience symptoms while others do not.

**American football players**. Of the 153 CTE cases, 63 had a history of participation in football, and the majority of football subjects played at the professional level. It is important to separate out this subgroup since former football players have dominated CTE in the modern era of CTE research. In the 2012 review of 68 cases of CTE, McKee et al. observed that pathological findings of CTE were significantly correlated to the number of years a subject participated in football [2]. However, despite the professional levels making up the majority of CTE cases, high school football players have also been reported to have CTE in spite of their limited mTBI exposure period. Although several of the high school football players were found to have CTE decades after their participation in high school football, three of the seven high school football players found to have CTE died prior to the age of 20. Despite this, the average age at death for football players is similar to the overall CTE cohort: 69% of CTE football cases compared to 75% of the remaining cases died prior to age 70. There was, however, a substantially higher prevalence of suicide in football players compared to the rest of the CTE cohort (17.5% (n = 11) vs. 5.26% (n = 3)). Football players account for 79% of all suicides case reports.

It is unknown whether this finding indicates that suicide is an at-risk behavior specifically related to former football players or if it is due to the bias associated with case reporting. Iverson et al. (2014) has noted that between the years 1960 and 2007 there were only nine cases of suicide of all former NFL players, again providing evidence that recent selective case reporting limits assertions as to risk factors associated with CTE [53].

Prevalence of substance abuse in American football players (n = 9; 14%) was lower compared to the rest of CTE cohort (n = 21; 23%). The limited data collected on substance abuse in the CTE literature prevents us to draw any firm conclusion about the association between CTE and substance abuse. However, it is of interest that the reported prevalence of substance abuse in former professional football players with CTE is substantially less than the prevalence suggested by other studies exploring substance abuse in former football players. For example, in a study by Cottler et al. (2011), 52% of retired football players used opioids during their NFL career with 71% reporting misuse [66]. What, if any, effect substance abuse contributes to CTE in football players remains unknown and requires further exploration.

Data on ApoE genotype was available for 53 out of the 63 football players (84.1%). A chi-square goodness of fit test was conducted to compare the prevalence of ApoE4 carriers in football mTBI subgroup with the general populations. The analysis revealed that the difference in frequency between the ApoE4 allele carriers in former football players with CTE and the general population was also non-significant (p = .56).

### Is there a CTE epidemic?

The data compiled in this review is considerably limited due to the inherent limitations associated with retrospective case reports used to study CTE for the last 80 years. Case reporting lacks consistency in the types of data reported in CTE cases, such as incomplete pre-morbid data and furthermore, CTE case reports are inherently subject to selection bias. Despite the many millions who have participated in contact sports and potentially suffered an mTBI, there are currently only 40 modern era CTE articles with 153 pathologically confirmed CTE cases reported.

Although CTE research is still in its early stages, especially with American football players, researchers are encouraged to move beyond case reporting. These pioneering reports suggest an association between chronic neurodegeneration changes and head trauma, but without prospective controlled studies definitive conclusions about the incidence, prevalence and associated risk factors cannot be drawn.

Based on this review, beyond a history of prior head trauma, no other evidence was found to be associated with CTE related brain changes. Other previously suggested risk factors for CTE, such as severity and frequency of pre-morbid mTBI, history of substance abuse, duration of play in a contact sport, underlying genetic factors or uniqueness of pre-morbid symptoms, cannot be meaningfully associated with CTE at this time.

Although prior head trauma has been considered a consistent risk factor for CTE, Hazrati et al. (2013) demonstrated not all of those with prior sports related head trauma will have later onset of neurological deficits or develop neuropathology associated with CTE. They observed that 3 out of 6 (50%) brains of former CFL athletes displayed CTE neuropathological abnormalities, although all 6 players had extensive histories of head injury [22]. Both Omalu et al. (2011) and McKee et al. (2012) observed similar findings in their cases where 6 out of 17 (35.3%) and 17 out of 85 (20%) subjects, respectively, were found negative for CTE neuropathologies, despite histories of multiple mTBI [2,3].

## Conclusion

Our comprehensive review of the medical literature revealed 153 unique cases of pathologically confirmed CTE cases reported as of August 1, 2013. This figure comes after accounting for a large number of duplicate cases in the literature (43% of all cases), which misrepresents the current extent of research and hence our understanding of CTE. Additionally, this figure represents a very small fraction of the total number of individuals in contact sports—in the many millions—who may have suffered concussions or other mTBI.

This review has provided some clarity as to the demographics of the CTE cases reported to date, specifically countering the belief that premature death is associated with the diagnosis of CTE [80,81]. This review provides evidence that the recent increase of deaths due to suicide and accidental death, which are prevalent in recent former professional football players found to have CTE, is responsible for the lower average age of death found in this cohort.

There remains a lack of clarity with regards to neuropathological findings and pre-morbid clinical findings which overlap with many common neurodegenerative diseases [62]. CTE researchers are currently attempting to develop classifications for symptom and degree of neuropathological severity in order to improve the diagnosis of CTE. This is important as it relates to age-related neurodegenerative changes.

The case reports of CTE thus far have not conclusively identified risk factors associated with the development of CTE beyond a history of brain trauma. Head injury severity, frequency and occurrence of an associated concussion have not been routinely documented in the literature and therefore are of unknown contribution. Some reports have suggested that more years playing professional football may increase risk, but this is suggestion has not been substantiated [2]. Preexisting genetic factors have been investigated as a possible contributing risk factor for the development of CTE. This review found that prevalence of ApoE4, a genetic marker for neurodegenerative diseases, is not significantly increased in the CTE cases to date compared to the general population [82,83].

Until recently, CTE was a pathological diagnosis primarily reported in former boxers. Since Omalu et al. reported the first CTE finding in a professional American football player in 2002 this formerly rare diagnosis has been thrust into a media, legal and research spotlight. Thus far, the described cases of CTE have exclusively been case reports or small series of cases. Several studies investigating small series of athletes with reported prior mTBI have found that although head trauma is a reported risk factor for CTE, not all individuals studied that experienced head trauma have been found to develop neuropathological changes associated with CTE [2,3,22].

This systematic review study emphasizes the need for further research into the epidemiology of CTE. Despite the lack of large scale systematic and randomized studies, the reporting of CTE in former professional American football players has led to wide spread speculation far beyond the conclusions that can be drawn based on the current state of CTE research. With CTE research in the early stages and the small number of current cases, there is no credible data with which to establish the incidence or prevalence of CTE in former contact sport participants.

## Supporting Information

S1 PRISMA Checklist PRISMA checklist for systematic review papers. (PDF)

S1 Table Complete overview of cases and demographics included in this systematic review. (DOCX)
