# Correction: Chronic Traumatic Encephalopathy in Contact Sports: A Systematic Review of All Reported Pathological Cases

**DOI:** 10.1371/journal.pone.0130507

**Published:** 2015-06-03

**Authors:** Joseph C. Maroon, Robert Winkelman, Jeffrey Bost, Austin Amos, Christina Mathyssek, Vincent Miele

The authors wish to amend the Competing Interests Statement for this article, which should have included additional information in relation to potential competing interests relevant to this work. The authors apologize for this omission and revise the Competing Interests Statement to read as below.

The University of Pittsburgh Medical Center has received grants from the National Football League and the Pittsburgh Steelers. Dr. Joseph Maroon is an unpaid consultant for the Pittsburgh Steelers football club. He has been the team neurosurgeon for the Pittsburgh Steelers since 1981 and the medical director for World Wrestling Entertainment Corporation since 2008 for the management of spine and brain-related injury. He also has served on the National Football League's Head, Neck and Spine Committee since 2007 and is currently a consultant to the committee. Dr. Maroon is a founder and shareholder in ImPACT (Immediate Post Concussion Assessment and Cognitive Testing), and the WWE has partnered with ImPACT to provide concussion management. Dr. Maroon has served as an expert witness in medical legal cases involving concussions. This does not alter the authors' adherence to PLOS ONE policies on sharing data and materials.

In addition, the authors would like to correct statements in the published article and provide additional clarification regarding the searches underlying the systematic review:

Figure 1 indicates that the authors conducted a meta-analysis on 40 studies, which is incorrect. The text in the last box of the flow chart should read "40 studies included in quantitative analysis." For the qualitative analysis of 58 studies, the authors gathered information on observed pathology, associated symptoms and suspected risk factors of chronic traumatic encephalopathy (CTE). The 18 qualitative studies that were excluded from the quantitative analysis did not present any pathologically confirmed case reports of CTE.

The following statement in the third paragraph of the Discussion section is incorrect: "This is also true for athletes of contact sports with risk for head impact but with no confirmed CTE cases to date, such as rugby or soccer players." There are both rugby and soccer players included in the authors' dataset of pathologically confirmed cases of CTE (Table S1).

The article reports a review of published data from 1954 to August 1, 2013. Because the review data collected did not have consistent demographic identifiers, each subject reported required an exhaustive process to cross-match the entire database to determine duplicate reporting and identify unique individuals. After the data collection, the analysis of the data and their preparation for publication took 12 months. Since the closing of the data collection in August 2013, there have been a number of additional reports documenting athletes and others with pathologically confirmed CTE. This includes an article by McKee and Robinson (2014) that reported on four US veterans from the recent wars who had a history of blast exposure and were found to have CTE at autopsy [1]. A systematic analysis of those cases, as well as larger studies, are warranted to improve our understanding of the epidemiology of CTE.

A related publication by McKee et al. (2014) published after the closure of our data collection [2]. McKee et al. assert that the presence of hyperphosphorylated tau in recently concussed young individuals suggests head injury and CTE are mechanistically linked. McKee et al. base this conclusion on the six individuals in their Table 1 that had a prior concussion and died soon after from suicide and OD. Of these six cases, the three who had hyperphosphorylated tau consistent with CTE, case IDs 1, 3, and 6, were also presented in McKee et al. 2013 as cases 36, 37, and 45 [3] and were included in the current review [4]. The assertion by McKee et al. is contrary to previous studies, including Smith et al. (2013), in which the authors concluded they did not believe the pathological features of CTE are associated with acute TBI [5]. Our study supports assertions by McKee et al. that CTE pathology is generally more advanced with increasing age and there is an increased incidence of suicide and accidental death in the select population being studied.
