# Supplementary material for: Chronic Traumatic Encephalopathy in Contact Sports: A Systematic Review of All Reported Pathological Cases
Source: PLoS One. 2015 Feb 11;10(2):e0117338. doi: 10.1371/journal.pone.0117338 (PMC4324991; doi:10.1371/journal.pone.0117338)
Supplement: S1 Table — (DOCX) [file pone.0117338.s002.docx]

Table S1: Complete overview of cases and demographics included in this systematic review

|  | **Source** | **mTBI exposure** | **Additional mTBI exposure risk** | **Age of death** | **ApoE** | **Type of death** | **Cause of death** | **Other diagnoses** | **Known history of substance abuse** |
| --- | --- | --- | --- | --- | --- | --- | --- | --- | --- |
|  | ***Football Players (n = 63)*** | |  |  |  |  |  |  |  |
| 1 | Omalu 2011^[3]^ | FB (HS) |  | 10-19 |  | A | TBI sequelae |  |  |
| 2 | Goldstein 2012^[20]^, McKee 2013^[2]^ | FB (HS) | BB (HS) | 10-19 |  | A | Second-impact Syndrom |  |  |
| 3 | Goldstein 2012^[20]^, McKee 2013^[2]^ | FB (HS) | Rugby | 10-19 | 33 | A | Cerebral oedema (Rugby) |  |  |
| 4 | Goldstein 2012^[20]^, McKee 2013^[2]^ | FB (HS) | Vet, IED | 20-29 | 33 | A | Intracranial Hemorrhaging |  |  |
| 5 | Goldstein 2012^[20]^, McKee 2013^[2]^ | FB (HS) | Vet | 20-29 | 34 | S | Suicide |  |  |
| 6 | McKee 2013^[2]^ | FB (HS) | Vet - IED, Prison guard | 30-39 | 33 | A | Overdose |  |  |
| 7 | McKee 2013^[2]^ | FB (HS) |  | 40-49 | 33 | S | Suicide |  |  |
| 8 | Goldstein 2012^[20]^, McKee 2013^[2]^ | FB (C) |  | 20-29 | 34 | S | Suicide |  |  |
| 9 | McKee 2013^[2]^ | FB (C) | WR (HS) | 30-39 | 33 | N | Respiratory failure | CTE-MND |  |
| 10 | McKee 2013^[2]^ | FB (C) |  | 40-49 | 33 | N | Respiratory failure | CTE-MND |  |
| 11 | McKee 2013^[2]^ | FB (C) |  | 50-59 | 33 | N | Malignancy |  |  |
| 12 | McKee 2013^[2]^ | FB (C) | Vet, BX, BB (HS) | 40-49 | 33 | N | Respiratory failure | CTE-MND |  |
| 13 | McKee 2013^[2]^ | FB (C) |  | 40-49 | 33 | A | Overdose |  | Y |
| 14 | McKee 2013^[2]^ | FB (C) |  | 60-69 | 44 | N | FTT | AD |  |
| 15 | McKee 2013^[2]^ | FB (C) |  | 60-69 | 33 | N | FTT | AD |  |
| 16 | McKee 2013^[2]^ | FB (C) | Vet, Rugby (C) | 60-69 | 33 | N | FTT | PD |  |
| 17 | Omalu 2005^[35]^; 2010^[33]^; 2011^[3]^ | FB (NFL) |  | 50-59 | 33 | N | Heart Attack |  | Y |
| 18 | Omalu 2006^[36]^; 2010^[33]^; 2011^[3]^ | FB (NFL) |  | 40-49 | 34 | S | Suicide |  | Y |
| 19 | McKee 2009^[5]^; 2010^[29]^; 2012^[2]^ | FB (NFL) |  | 40-49 | 44 | A | Gunshot wound |  | Y |
| 20 | Omalu 2010^[37]^; 2010^[34]^; 2011^[3]^ | FB (NFL) |  | 40-49 | 33 | S | Suicide |  | Y |
| 21 | Omalu 2010^[33]^; 2011^[3]^ | FB (NFL) |  | 30-39 |  | S | Suicide |  | Y |
| 22 | Omalu 2011^[3]^ | FB (NFL) |  | 50-59 | 33 | N | Liver and Pancreatitis |  |  |
| 23 | Omalu 2011^[3]^ | FB (NFL) |  | 30-39 |  | A | Overdose |  | Y |
| 24 | Media^[47]^ | FB (NFL) |  | 20-29 |  | A | TBI Sequelae |  | Y |
| 25 | Media^[48]^ | FB (NFL) |  | 60-69 |  | S | Suicide |  |  |
| 26 | Media^[49]^ | FB (NFL) |  | 40-49 |  | S | Suicide |  |  |
| 27 | McKee 2013^[2]^ | FB (NFL) |  | 20-29 | 33 | S | Suicide |  |  |
| 28 | McKee 2013^[2]^ | FB (NFL) |  | 30-39 | 33 | N | Cardiac |  |  |
| 29 | McKee 2013^[2]^ | FB (NFL) |  | 50-59 | 34 | N | Malignancy |  |  |
| 30 | McKee 2010^[29]^; 2012^[2]^ | FB (NFL) |  | 40-49 | 33 | N | Cardiac |  |  |
| 31 | McKee 2010^[29]^; 2012^[2]^ | FB (NFL) |  | 40-49 | 34 | N | Respiratory failure | CTE-MND |  |
| 32 | McKee 2010^[29]^; 2012^[2]^ | FB (NFL) | Vet | 80-89 | 33 | N | Cardiac |  |  |
| 33 | McKee 2013^[2]^ | FB (NFL) |  | 30-39 | 33 | S | Suicide |  |  |
| 34 | McKee 2010^[29]^; 2012^[2]^ | FB (NFL) |  | 40-49 | 44 | A | Overdose |  | Y |
| 35 | McKee 2013^[2]^ | FB (NFL) |  | 50-59 | 34 | S | Suicide |  |  |
| 36 | McKee 2013^[2]^ | FB (NFL) |  | 50-59 | 34 | N | Cardiac |  |  |
| 37 | McKee 2010^[29]^; 2012^[2]^ | FB (NFL) |  | 60-69 | 23 | N | Respiratory Failure | CTE-MND |  |
| 38 | McKee 2013^[2]^ | FB (NFL) |  | 60-69 | 33 | A | Overdose |  |  |
| 39 | McKee 2013^[2]^ | FB (NFL) |  | 60-69 | 33 | N | Cardiac |  |  |
| 40 | McKee 2013^[2]^ | FB (NFL) |  | 60-69 | 33 | N | Respiratory Failure | CTE-MND |  |
| 41 | McKee 2013^[2]^ | FB (NFL) |  | 70-79 | 34 | N | Malignancy |  |  |
| 42 | McKee 2013^[2]^ | FB (NFL) |  | 70-79 | 23 | N | Cardiac |  |  |
| 43 | McKee 2013^[2]^ | FB (NFL) | Vet | 80-89 | 44 | N | Respiratory Failure |  |  |
| 44 | McKee 2013^[2]^ | FB (NFL) | Vet | 70-79 | 33 | N | Respiratory Failure |  |  |
| 45 | McKee 2010^[29]^; 2012^[2]^ | FB (NFL) |  | 80-89 | 33 | N | FTT |  |  |
| 46 | McKee 2013^[2]^ | FB (NFL) | Vet | 80-89 | 33 | N | FTT |  |  |
| 47 | McKee 2013^[2]^ | FB (NFL) | Vet | 90-99 | 33 | N | FTT |  |  |
| 48 | McKee 2013^[2]^ | FB (NFL) | Vet | 60-69 | 23 | N | FTT | AD |  |
| 49 | McKee 2013^[2]^ | FB (NFL) | Vet | 70-79 | 34 | N | FTT | AD |  |
| 50 | McKee 2013^[2]^ | FB (NFL) |  | 60-69 | 33 | N | FTT | AD +PD |  |
| 51 | McKee 2013^[2]^ | FB (NFL) |  | 70-79 | 34 | N | FTT | AD + LBD |  |
| 52 | McKee 2013^[2]^ | FB (NFL) |  | 60-69 | 24 | N | Respiratory Failure | LBD |  |
| 53 | McKee 2013^[2]^ | FB (NFL) |  | 70-79 | 33 | N | Cardiac | LBD |  |
| 54 | McKee 2013^[2]^ | FB (NFL) | Vet | 70-79 | 44 | N | Cardiac | PD |  |
| 55 | McKee 2013^[2]^ | FB (NFL) | Vet | 80-89 | 33 | N | Malignancy | LBD |  |
| 56 | McKee 2013^[2]^ | FB (NFL) |  | 70-79 | 33 | N | Cardiac | PD + FTLD-TDP |  |
| 57 | McKee 2013^[2]^ | FB (NFL) |  | 60-69 | 33 | N | FTT | Pick's disease |  |
| 58 | McKee 2013^[2]^ | FB (NFL) |  | 80-89 | 34 | N | FTT | FTLD - TDP |  |
| 59 | Hazrati 2013^[22]^ | FB (CFL) |  | 70-79 |  | N | FTT | PD + LBD |  |
| 60 | Hazrati 2013^[22]^ | FB (CFL) |  | 60-69 |  | N | Malignancy |  |  |
| 61 | Hazrati 2013^[22]^ | FB (CFL) |  | 80-89 |  | N | FTT | AD |  |
| 62 | McKee 2013^[2]^ | FB (CFL) |  | 70-79 | 23 | N | Respiratory Failure | PD + PSP |  |
| 63 | McKee 2013^[2]^ | FB (SP) | Vet | 80-89 | 33 | N | FTT |  |  |
|  | ***Boxing (n = 69)*** |  |  |  |  |  |  |  |  |
| 64 | Brandenburg 1954^[11]^, McKee 2009^[5]^ | BX (AM) |  | 50-59 |  | N | Cerebral Hemorrhaging |  |  |
| 65 | Grahmann 1957^[21]^, McKee 2009^[5]^ | BX (AM) |  | 40-49 |  | N | Stroke |  |  |
| 66 | Neubuerger 1959^[30]^, McKee 2009^[5]^ | BX (AM) |  | 50-59 |  |  |  |  |  |
| 67 | Neubuerger 1959^[30]^, McKee 2009^[5]^ | BX |  | 50-59 |  | N | Progressive Pulmonary Insuffiency |  |  |
| 68 | Corsellis 1959^[13]^ | BX |  | 60-69 |  |  |  |  |  |
| 69 | Corsellis 1959^[13]^; 1973^[14]^, Roberts 1990^[41]^, Dale 1991^[17]^, McKee 2009^[5]^ | BX (AM) | Vet, Bike Accident | 50-59 |  | N | Pneumonia |  |  |
| 70 | Courville 1962^[16]^, McKee 2009^[5]^ | BX |  | 40-49 |  | N | Episode of Hypoglycemia |  |  |
| 71 | Mawdsley 1963^[28]^, McKee 2009^[5]^ | BX |  |  |  |  |  |  |  |
| 72 | Mawdsley 1963^[28]^, McKee 2009^[5]^ | BX |  |  |  |  |  |  |  |
| 73 | Constantinidis 1967^[12]^, Hof 1992^[23]^, McKee 2009^[5]^, Costanza 2011^[15]^ | BX |  | 50-59 |  | N | Respiratory Failure |  |  |
| 74 | Payne 1968^[39]^, McKee 2009^[5]^ | BX (P) |  |  |  | N | Coronary Artheroma |  |  |
| 75 | Payne 1968^[39]^, McKee 2009^[5]^ | BX (P) | Motor Cycle Accident | 40-49 |  | N | Heart Failure Due to Hypertension |  |  |
| 76 | Payne 1968^[39]^, McKee 2009^[5]^ | BX (P) | Vet | 40-49 |  | N | Acute Interstitial Pneumonia |  | Y |
| 77 | Payne 1968^[39]^, McKee 2009^[5]^ | BX (P) |  | 40-49 |  | N | Myocardial Infarction |  | Y |
| 78 | Payne 1968^[39]^, McKee 2009^[5]^ | BX |  | 40-49 |  | N | Coronary Artheroma |  |  |
| 79 | Payne 1968^[39]^, McKee 2009^[5]^ | BX | Vet | 20-29 |  | A | Stab Wound to the Heart |  |  |
| 80 | Corsellis 1973^[14]^, Roberts 1990^[41]^, Tokuda 1991^[44]^, McKee 2009^[5]^ | BX (P) | 3 MVA | 60-69 |  | N | Malignancy |  | Y |
| 81 | Corsellis 1973^[14]^, Roberts 1990^[41]^, Allsop 1990^[9]^, Dale 1991^[17]^, Tokuda 1991^[44]^, McKee 2009^[5]^ | BX (P) |  | 70-79 |  | N | FTT |  |  |
| 82 | Corsellis 1973^[14]^, Roberts 1990^[41]^, Allsop 1990^[9]^, Dale 1991^[17]^, McKee 2009^[5]^ | BX (P) |  | 60-69 |  | N | Pneumonia |  | Y |
| 83 | Corsellis 1973^[14]^, Roberts 1990^[41]^, Allsop 1990^[9]^, Dale 1991^[17]^, McKee 2009^[5]^ | BX (P) | Vet | 60-69 |  | N | Infection |  | Y |
| 84 | Corsellis 1973^[14]^, Roberts 1990^[41]^, Tokuda 1991^[44]^, McKee 2009^[5]^ | BX (P) |  | 60-69 |  | N | Pneumonia/Cardiac Ischemia |  | Y |
| 85 | Corsellis 1973^[14]^, Roberts 1990^[41]^, Dale 1991^[17]^, Tokuda 1991^[44]^, McKee 2009^[5]^ | BX (P) |  | 80-89 |  | N | Pneumonia |  |  |
| 86 | Corsellis 1973^[14]^, Roberts 1990^[41]^, Allsop 1990^[9]^, Dale 1991^[17]^, McKee 2009^[5]^ | BX (P) |  | 60-69 |  | N | Pneumonia |  | Y |
| 87 | Corsellis 1973^[14]^, Roberts 1990^[41]^, Tokuda 1991^[44]^, McKee 2009^[5]^ | BX (P) |  | 70-79 |  |  |  |  |  |
| 88 | Corsellis 1973^[14]^, Roberts 1990^[41]^, McKee 2009^[5]^ | BX (P) | Vet | 70-79 |  | N | FTT/Pneumonia |  | Y |
| 89 | Corsellis 1973^[14]^, Roberts 1990^[41]^, McKee 2009^[5]^ | BX (P) |  | 60-69 |  | N | FTT |  | Y |
| 90 | Corsellis 1973^[14]^, Roberts 1990^[41]^, Dale 1991^[17]^, McKee 2009^[5]^ | BX (P) | Vet | 60-69 |  | N |  |  |  |
| 91 | Corsellis 1973^[14]^, Dale 1991^[17]^, McKee 2009^[5]^ | BX (P) |  | 90-99 |  | N | Cardiac Ischemia |  |  |
| 92 | Corsellis 1973^[14]^, Roberts 1990^[41]^, Allsop 1990^[9]^, Dale 1991^[17]^, McKee 2009^[5]^ | BX (AM) | Vet | 60-69 |  | N | Cerebral Hemorrhage |  |  |
| 93 | Corsellis 1973^[14]^, Roberts 1990^[41]^, Allsop 1990^[9]^, Dale 1991^[17]^, McKee 2009^[5]^ | BX (AM) | Vet | 50-59 |  | A | Car Accident - Skull Fracture/Cerebral Hemorrhage |  |  |
| 94 | Mann 1983^[27]^ | BX |  | 50-59 |  |  |  |  |  |
| 95 | Mann 1983^[27]^ | BX |  | 50-59 |  |  |  |  |  |
| 96 | Mann 1983^[27]^ | BX |  | 50-59 |  |  |  |  |  |
| 97 | Mann 1983^[27]^ | BX |  | 60-69 |  |  |  |  |  |
| 98 | Roberts 1990^[41]^ | BX (P) |  | 20-29 |  |  |  |  |  |
| 99 | Roberts 1990^[41]^ | BX (P) |  | 50-59 |  |  |  |  |  |
| 100 | Roberts 1990^[41]^ | BX (P) |  | 60-69 |  |  |  |  |  |
| 101 | Roberts 1990^[41]^ | BX (P) |  | 60-69 |  |  |  |  |  |
| 102 | Roberts 1990^[41]^ | BX (AM) |  | 60-69 |  |  |  |  |  |
| 103 | Roberts 1990^[41]^ | BX (AM) |  | 70-79 |  |  |  |  |  |
| 104 | Dale 1991^[17]^ | BX (P) |  | 60-69 |  |  |  |  |  |
| 105 | Dale 1991^[17]^ | BX (P) |  | 60-69 |  |  |  |  |  |
| 106 | Dale 1991^[17]^ | BX (P) |  | 60-69 |  |  |  |  |  |
| 107 | Dale 1991^[17]^ | BX (AM) |  | 60-69 |  |  |  |  |  |
| 108 | Dale 1991^[17]^ | BX (AM) |  | 70-79 |  |  |  |  |  |
| 109 | Tokuda 1991^[44]^ | BX |  | 70-79 |  |  |  |  |  |
| 110 | Hof 1992^[23]^, McKee 2009^[5]^ | BX (P) |  | 60-69 |  |  |  |  |  |
| 111 | Hof 1992^[23]^, McKee 2009^[5]^ | BX (P) |  | 60-69 |  |  |  |  |  |
| 112 | Jordan 1995^[25]^, McKee 2009^[5]^ | BX |  | 70-79 | 34 | A | TBI sequelae |  |  |
| 113 | Geddes 1996^[19]^; 1999^[18]^, McKee 2009^[5]^ | BX (P) |  | 20-29 |  | A | TBI sequelae |  |  |
| 114 | Geddes 1999^[18]^, McKee 2009^[5]^ | BX (AM) |  | 20-29 |  | N | Death during seizure |  |  |
| 115 | Newell 1999^[31]^, Schmidt 2001^[43]^, McKee 2009^[5]^ | BX (P) |  | 60-69 | 34 |  |  |  | Y |
| 116 | Schmidt 2001^[43]^, McKee 2009^[5]^ | BX |  | 70-79 |  | N | FTT |  |  |
| 117 | Areza 2007^[10]^, McKee 2009^[5]^ | BX (P) |  | 60-69 | 33 | N | Malignancy |  |  |
| 118 | McKee 2009^[5]^; 2010^[29]^; 2013^[2]^ | BX (P) | Vet | 70-79 | 33 | N | FTT |  |  |
| 119 | McKee 2009^[5]^; 2010^[29]^; 2013^[2]^ | BX (P) |  | 80-89 | 34 | N | Sepsis |  | Y |
| 120 | Nowak 2009^[32]^ | BX (P) |  | 70-79 | 24 | N |  |  |  |
| 121 | King 2010^[26]^ | BX (P) |  | 70-79 |  |  |  |  |  |
| 122 | King 2010^[26]^ | BX (P) |  | 60-69 |  |  |  |  |  |
| 123 | King 2010^[26]^ | BX (P) |  | 60-69 |  |  |  |  |  |
| 124 | McKee 2010^[29]^ | BX (P) |  | 70-79 |  |  |  |  |  |
| 125 | McKee 2010^[29]^; 2013^[2]^ | BX (P) |  | 60-69 | 33 | N | Cardiac | CTE-MND | Y |
| 126 | Omalu 2011^[3]^ | BX (P) |  | 50-59 | 33 |  |  |  |  |
| 127 | Saing 2012^[42]^ | BX (P) |  | 50-59 | 34 | N | FTT |  | Y |
| 128 | McKee 2013^[2]^ | BX (P) |  | 40-49 | 33 | S | Suicide |  |  |
| 129 | McKee 2013^[2]^ | BX (P) |  | 50-59 | 33 | N | Respiratory Failure |  |  |
| 130 | McKee 2013^[2]^ | BX (P) |  | 60-69 | 34 | N | Respiratory Failure |  |  |
| 131 | McKee 2013^[2]^ | BX (P) | Vet | 70-79 | 33 | N | FTT |  |  |
| 132 | McKee 2013^[2]^ | BX (AM) | Vet | 90-99 |  | N | FTT |  |  |
|  | ***Hockey Players (n = 5)*** | |  |  |  |  |  |  |  |
| 133 | McKee 2010^[29]^; 2012^[2]^ | HK (P) |  | 70-79 | 34 | N | FTT | LBD | Y |
| 134 | McKee 2013^[2]^ | HK (P) |  | 20-29 | 33 | A | Overdose |  | Y |
| 135 | McKee 2013^[2]^ | HK (P) |  | 40-49 | 33 | N | Cardiac |  | Y |
| 136 | McKee 2013^[2]^ | HK (P) |  | 50-59 | 34 | N | Cardiac |  |  |
| 137 | McKee 2013^[2]^ | HK (AM) | Vet | 80-89 | 33 | N | FTT | AD + PD |  |
|  | ***Wrestling (n = 3)*** |  |  |  |  |  |  |  |  |
| 138 | McKee 2009^[5]^, Omalu 2010^[33,37]^; 2011^[3]^ | WR (P) |  | 40-49 | 33 | S | Suicide |  | Y |
| 139 | Omalu 2011^[3]^ | WR (P) |  | 30-39 | 34 | A | Overdose |  | Y |
| 140 | Goldstein 2012^[20]^, McKee 2013^[2]^ | WR (P) |  | 20-29 | 33 | A | Overdose |  |  |
|  | ***Veterans (n = 5)*** |  |  |  |  |  |  |  |  |
| 141 | Media^[46]^ | Vet |  | 60-69 |  | N | Cardiac |  | Y |
| 142 | Omalu 2011^[38]^ | Vet |  | 20-29 | 34 | S | Suicide |  | Y |
| 143 | Goldstein 2012^[20]^ | Vet | IED | 30-39 |  | A | Overdose |  |  |
| 144 | Goldstein 2012^[20]^, McKee 2013^[2]^ | Vet | IED, MVA | 40-49 | 33 | N | Cerebral Aneurysm |  |  |
| 145 | McKee 2013^[2]^ | Vet | MVA, altercation | 80-89 | 33 | N | Pneumonia |  |  |
| 146 | McKee 2013^[2]^ | Vet | TBI, PT Epilepsy | 70-79 | 33 | N | Pneumonia |  |  |
|  | ***Miscellaneous (n =7)*** | |  |  |  |  |  |  |  |
| 147 | Roberts 1990^[40]^, McKee 2009^[5]^ | Physical Abuse |  | 70-79 |  |  |  |  |  |
| 148 | Hof 1991^[24]^, McKee 2009^[5]^ | Autistic Head Banging |  | 20-29 |  |  |  |  |  |
| 149 | Williams 1996^[45]^, McKee 2009^[5]^ | Circus Clown |  | 30-39 |  |  |  |  | Y |
| 150 | Geddes 1999^[18]^, McKee 2009^[5]^ | Epilepsy |  | 20-29 | 33 |  |  |  |  |
| 151 | Geddes 1999^[18]^, McKee 2009^[5]^ | Head banging |  | 20-29 |  | A | TBI Sequealae |  |  |
| 152 | Geddes 1999^[18]^, McKee 2009^[5]^ | Soccer |  | 20-29 | 33 | A | TBI Sequealae |  |  |
| 153 | McKee 2013^[2]^ | Self-Injury |  | 50-59 |  | N | Respiratory Failure |  |  |

FB=Football; Vet=Veteran; BB=Baseball; BX=Boxing, HK=Hockey; WR=Wrestling; AM=Amateur; P=Professional; HS=High School; C=College; NFL=National Football League; CFL=Canadian Football League; SP=Semi-Professional; MVA= Motor Vehicle Accident; IED=Improvised Explosive Device; TBI=Traumatic Brain Injury; PT=Post-traumatic; A=Accidental; N=Natural; S=Suicide; FTT=Failure to thrive; CTE-MND=Chronic Traumatic Encephalopathy- Motor neuron disease ; AD=Alzheimer’s disease; PD=Parkinson’s disease; LBD=Lewy Body Dementia; FTLD-TDP= Frontotemporal lobar degeneration- TAR-DNA binding protein; PSP= Progressive supranuclear palsy; Y=Yes.
